# Applications of Different Weighting Schemes to Improve Pathway-Based Analysis

**DOI:** 10.1155/2011/463645

**Published:** 2011-05-22

**Authors:** Sook S. Ha, Inyoung Kim, Yue Wang, Jianhua Xuan

**Affiliations:** ^1^Bradley Department of Electrical and Computer Engineering, Virginia Tech, Arlington, VA 22203, USA; ^2^Department of Statistics, Virginia Tech, Blacksburg, VA 24061, USA

## Abstract

Conventionally, pathway-based analysis assumes that genes in a pathway equally contribute to a biological function, thus assigning uniform weight to genes. However, this assumption has been proved incorrect, and applying uniform weight in the pathway analysis may not be an appropriate approach for the tasks like molecular classification of diseases, as genes in a functional group may have different predicting power. Hence, we propose to use different weights to genes in pathway-based analysis and devise four weighting schemes. We applied them in two existing pathway analysis methods using both real and simulated gene expression data for pathways. Among all schemes, random weighting scheme, which generates random weights and selects optimal weights minimizing an objective function, performs best in terms of *P* value or error rate reduction. Weighting changes pathway scoring and brings up some new significant pathways, leading to the detection of disease-related genes that are missed under uniform weight.

## 1. Introduction

With the advent of microarray technology in the field of biomedical research [1–7], numerous statistical methods [8, 9] were proposed to analyze microarray gene expression data. But most are single gene based and do not consider the interacting relationship or dependencies among genes in a functional group. In single gene-based analysis, most subtly but coordinated differentially expressed genes are often not identified as significant and usually dropped by a strict cutoff threshold feature selection [10, 11]. In contrast, pathway-based analysis considers a set of biologically related genes and helps detect subtle changes in gene expression with the help of a joint effort by genes [3, 4, 12, 13]. Many researchers discussed the advantages of pathway-based analysis. Subramanian, for instance, considered an enrichment-based approach using various Kolmogorove-Smirnov statistics [3]; Curtis gave a good review of computational approaches proposed for pathway-based analysis [4]; Goeman et al. proposed the global test based on a generalized linear model [12]; Pang et al. described the random forest-based pathway analysis [13]; Harris et al. considered gene grouping based on gene ontology [14]; Misman et al. provide good reviews on those in [15]. 

A biological pathway is a series of actions among molecules in a cell that leads to a certain product or a change in a cell. Such a pathway can trigger the assembly of new molecules, such as fat or protein. Pathways can also turn genes on and off or spur a cell to move [16]. Biological pathways help researchers learn a lot about human disease, since identifying genes, proteins, and other molecules involved in a biological pathway can provide clues about what goes wrong when a disease strikes. Researchers may compare certain biological pathways in a healthy person to the same pathways in a person with a disease to discover the roots of the disorder. Using pathways extensively allows a quick overview of expression results in relation to biological mechanisms, facilitating the understanding of gene, protein, and metabolite interactions at higher levels. Over the past decade, researchers have discovered many important biological pathways through laboratory studies of cultured cells and various organisms, and they are stored in public domain biological pathway databases [16]. Biological pathways have been also curated manually combining three content sources: public domain databases, literature, and experts [17]. 

Pathway analysis aims to define the meaning of biological processes by identifying significant pathways through statistical evaluations. Pathways are scored in statistical evaluations based on activity, coregulation, and cascade effects in pathways as measured by the gene expression levels from the microarray experimental data. This score will rank those pathways higher in which more genes are overexpressed or underexpressed with reference to reference state [18]. Ranking pathways relevant to a particular biological process or disease is useful, since it allows researchers to focus on a smaller number of pathways for further study of the biological process or disease of interest. Most pathway analysis tools and methods, however, are assuming that all genes in a pathway are equally contributing to a biological process, and thus assigning uniform weight. But this assumption has been proved incorrect [19] because some genes may have higher relevancies to a particular biological process, and those genes presumably have higher predicting or classifying power than the others. One issue in the pathway analysis is the quality of pathways since biological pathway databases are not comprehensive, and the biological pathway content varies greatly in quality and completeness among the tools and databases [17]. Pathway data taken from public databases and open literatures may include nonrelevant genes and/or exclude relevant genes [20]. For instance, in the case of the famous Mootha's type II diabetes pathway dataset [21], genes such as CAP1, MAPP2K6, ARF6, and SGK contained in the pathway ID 36, c17 U133 probes, are known to be related to human insulin signaling [15], while other genes are not yet. Also, SHC contained in the pathway ID 229 is known to be related to human insulin signaling, while others are not yet. 

To address the problem of pathway quality and incompleteness in the pathway analysis tools and approaches, some researchers tried to minimize the misspecifications by defining signature genes to represent pathway behaviors, and/or refining pathways to adapt to specific conditions by removing unaltered genes from the dataset [19, 22–24]. Others tried to improve the functional interpretation of gene groups by including additional information associated with the group [24]. Joining such efforts, we propose to apply nonuniform weighting scheme, which applies different weights to the genes in a pathway-based on the relevancies of genes to a related biological process or disease. The intuitive ideas behind our proposed ideas are that not all genes grouped in a pathway are related to a particular biological process or disease with the same significance, and thus applying weight to the genes proportional to their relevancies to a certain biological process or disease may generate more accurate results for pathway based analysis such as the molecular classification of diseases. 

To investigate the impact of using weighting schemes in pathway based analysis, we devise four different weighting schemes and incorporate them into the existing pathway analysis methods, such as the global test [12] and the random forests [13, 25–27]. Our schemes essentially apply larger weights to more differentially expressed genes between different sample groups (i.e., normal vs. tumor samples), so those genes impact more on the final results of analysis. The four weighting schemes we introduce in this paper are as follows. The first weighting scheme is based on the absolute value of two sample *t* statistics denoted as *absT*. The second one is based on the *Q* test statistic of the global test denoted as *Qdiff*. The third and the fourth ones are based on a computational approach, which assigns weights randomly to genes and selects optimal weights minimizing an objective function. The third scheme called *RWV* (*random weight vector*) is to assign *m* weights for *m* genes, in which all samples of a gene are assigned the same weight. The fourth one called *RWM* (*random weight matrix*) is to assign a matrix of weights for a pathway of *m*(*genes*) × *n*(*samples*), in which samples of a gene are assigned different weights.

We performed our experiments using the type II diabetes dataset obtained from Mootha et al. [28] and the canine dataset from Enerson et al. [29]. We also used simulated datasets to gain an in-depth understanding of weighting effects in a controlled way. In our experiments, we apply each weighting scheme onto the datasets and select top 20 or 33 significant pathways. We evaluate the performance of our weighting schemes by comparing the *P* values of the pathways selected using each scheme with those selected using uniform weighting. We observe that when our weights are applied, the scoring of the pathways is changed, and some pathways originally in lower ranks are elevated to higher ranks, hence, contributing to improved prediction rates. According to the previous studies [28, 30–34], several significant pathways identified by our weighting schemes are biophysiologically associated with related diseases.

## 2. Materials and Methods

### 2.1. Global Test and Random Forest

We used the global test [12] and the random forests [13, 25, 26] methods to investigate the impact of weighting in the pathway-based analysis and to evaluate the performance of our weighing schemes. First, we review the two methods briefly to explain how we incorporate our proposed weighting schemes into these methods.

#### 2.1.1. Overview of Global Test

The global test method is a pathway analysis method developed by Goeman et al. [12]. It tests whether subjects with similar gene expression profiles have similar class labels, based on a logistic regression. Suppose that gene expression data containing *n* samples for *p* genes is normalized. Of these *p* genes, a subgroup of *m*  (1 ≤ *m* ≤ *p*) genes is to be tested. Let *X* = (*x*_*ij*_) be an *n* × *m* data matrix containing *m* genes for *n* samples of interest, and *Y*_*i*_ as the clinical outcome of the *i*_th_ sample (*n* × 1vector). To model how the clinical outcome *Y* depends on the gene expression data  *X*, the global test adopts the generalized linear model framework developed by McCullagh [35], expressed as follows:

(1) $$E\left(Y_{i}\;|\;\vec{\beta }\right)=h^{-1}\left(\alpha +\overset{m}{\underset{j=1}{\sum }}x_{ij}\beta_{j}\right),$$

where *β*_*j*_ is the regression coefficient for gene *j*  (*j* = 1,…, *m*), *h* is a link function (e.g., the logit function), and *α* is an intercept. Testing a predictive effect of the gene expressions on the clinical outcome is equivalent to testing the hypothesis *H*_0_ : *β*_1_ = *β*_1_ = *β*_2_ ⋯ = *β*_*m*_ = 0. Assume that *β*_1_,…, *β*_*m*_ are a sample from some common distribution with zero mean and variance *τ* ^2^, then a single unknown parameter *τ* ^2^ determines the allowed deviation of the regression coefficients from zero. Thus, the null hypothesis is *H*_0_ : *τ* ^2^ = 0. The formula *r*_*i*_ = ∑_*j*_ *x*_*ij*_ *β*_*j*_  (*i* = 1,…, *n*) is the linear predictor, that is, the total effect of all covariates for the *i*_th_ sample. As *r* = (*r*_1_,…, *r*_*n*_) is a random vector with *E*(*r*) = 0 and cov (*R*) = *τ* ^2^ *XX*′, the generalized linear model is simplified to *E*(*Y*_*i*_ | *β*) = *h* ^−1^(*α* + *r*_*i*_). A test statistic for testing *H*_0_ is defined as

(2) $$Q=\frac{1}{\mu^{2}}\overset{n}{\underset{i=1}{\sum }}\overset{m}{\underset{j=1}{\sum }}R_{ij}\left(Y_{i}-\mu \right)\left(Y_{j}-\mu \right),$$

where *R* = (1/*m*)*XX*′ is an *n* × *n* matrix proportional to the covariance matrix of the random effects *r*, *μ* = *h* ^−1^(*α*) is the expectation of *Y* under *H*_0_, and (*Y* − *μ*)(*Y*−*μ*)′ is the covariance matrix of the clinical outcomes of the samples. The test statistic *Q* has a higher value if the terms of the two matrices are correlated more. Essentially, it tests whether samples with similar gene expressions also have similar outcomes. The empirical distribution of test statistic *Q* under the null hypothesis *H*_0_ is calculated across all samples by randomly taking a large number of permutations (such as 100,000) of the vector *Y*from the outcomes. The empirical *P* value is the frequency such that *Q* for the permuted *Y* is at least as large as the true *Q*, divided by the number of permutations. For our microarray datasets cases, *Y* is 1 for a disease sample or 0 for a normal sample. 

The reason we selected the global test pathway analysis method for our study of weighting effect in the pathway-based analysis is that the generation and the assignment of weights for the genes in a pathway is easy and straightforward in the global test. Multiplying a desired nonuniform weight matrix *W* = (*w*_*ij*_) to a gene expression data matrix *X* = (*x*_*ij*_) in the global test method does not incur any side effects.

### 2.2. Overview of Random Forests

The random forests are a tree-based method developed by Breiman et al. (1984, 2001) [25–27], which can be used for classifications or regressions [13]. The method grows multiple classification or regression trees using a deterministic algorithm, in which each tree is constructed using a different bootstrap sample from the original data. It leaves about one-third of the cases out of the bootstrap (out-of-bag) samples for testing purpose. The out-of-bag (OOB) samples are not used in constructing the *k*_th_ tree but saved to be used as a test set. At the end of the run, it takes the *i*_th_ sample to be the class which receives most of the votes every time case *n* is the out of bag. The proportion of times that *i* is not equal to the true class of *n* averaged over all cases is called the estimated out-of-bag (OOB) error (http://stat-www.berkeley.edu/). Pang et al. [13] are the first group who proposed to apply the random forests approach to pathway analysis, and we adopted their approach to study the weighting effect in the pathway analysis. Our objective is to find the optimal weight W* that minimizes the OOB error rate using the objective function we modify in the following:

(3) $$W^{*}=\underset{w}{\text{argmin}}\;\left[F\left(wX\right)\right],$$

where *F*(*X*) is the original cost function of the random forests that computes the OOB error rate of a group of data *X*, and *w* is a weight matrix for the group of data *X*. Our objective is to find the weight matrix *w* which minimizes the estimated OOB classification error for each pathway.

### 2.3. Proposed Weighting Schemes

We considered four nonuniform weight schemes, which intend to generate the weight for each gene in a pathway, based on its degree of differential expression between the different phenotypes. In this section, we describe the rationale behind each weighting scheme, generation, and assignment of nonuniform weights for genes in a pathway.

#### 2.3.1. *absT* Based on Two-Sample *t*-Test Statistic |*T*|

The two-sample *t*-test statistic is widely used to determine if the means of two populations are equal [35]. To measure how differentially a gene is expressed between two different groups (i.e., normal versus disease), we calculate the two-sample *t*-test statistic of the gene and take the absolute value of it and denote it as |*T*|. The *absT* scheme determines the weight of each gene in a pathway using the |*T*| value of each gene divided by the sum of all |*T*| values of all genes in the pathway. Mathematically, the weight for the *j*_th_ gene *W* ^|*T*|^_(*j*)_ is expressed in the following formula:

(4) $${W^{\left|T\right|}}_{\left(j\right)}=\frac{\left|T_{\left(j\right)}\right|}{\sum_{j=1}^{m}\left|T_{\left(j\right)}\right|}.$$

With this scheme, the most differentially expressed gene will have the largest |*T*| value and get the largest weight. The rationale is based on the hypothesis that more differentially expressed genes have higher relevancy to the disease or the phenotype of interest.

#### 2.3.2. *Qdiff* Based on the Test Statistic *Q* of the Global Test

The test statistic *Q* of the global test is a test to find whether samples with similar gene expressions also have similar outcomes. If the covariance structure of the gene expressions between two sample groups resembles the covariance structure of their outcomes, the *Q* statistics is large. The proposed *Qdiff *weighting scheme uses the *Q* statistic of a pathway to construct the weights for genes in the pathway. The idea is based on our hypothesis that if excluding one gene from a pathway results in a large difference in the original test statistic *Q*, the excluded gene may have a strong relevancy to the related disease or phenotype. To determine the weight for the *j*_th_ gene in a pathway containing *m* genes, the scheme uses the following formula:

(5) $${W^{\left|Q_{diff}\right|}}_{\left(j\right)}=\frac{\left|Q-Q_{\left(-j\right)}\right|}{\sum_{j=1}^{m}\left|Q-Q_{\left(-j\right)}\right|}.$$

Here, *Q* is the test statistic of the pathway including all *m* genes, and *Q*_(−*j*)_ is the test statistic of the same pathway but excluding the *j*_th_ gene. The weight of the *j*_th_ gene is determined by the difference of these two test statistics *Q* and *Q*_(−*j*)_, divided by the sum of all such differences calculated for all *m* genes in the pathway.

#### 2.3.3. *RWV* Based on Random Weight Vectors Generated by a Computational Approach

The computational *RWV *(*random weight vector*) scheme assigns *m* random weights to *m* genes in a pathway and identifies the optimal *m* weights vector minimizing the *P* value of the pathway. It uses the following pseudocode algorithm to obtain the optimal *m* weights vector for each pathway.

**Step 1**

Run the global test on the original gene expression of a pathway and obtain the *P*-value for the pathway. Initialize this *P*-value as *minP* and the uniform weight vector as *optW. *

**Step 2**

for *i* = 1: COUNT.

**Substep 1**

Generate a set of *m* random values in the pre-defined range (i.e., 0.1 ≤  range ≤1.0).

**Substep 2**

Pick *m* values randomly from the set of *m* random values constructed in Substep 1, allowing replacements.

**Substep 3**

Multiply each gene expression *X*_*j*_ = [*X*_*j*,1_, *X*_*j*,2_,…, *X*_*j*,*n*_]with the corresponding weight *w*_*j*_  (1 ≤ *j* ≤ *m*). This process constructs a weighted gene expression matrix *wX* for the pathway

(6) $$wX=\left[\begin{array}{c} w_{1}X_{1}\\ w_{2}X_{2}\\ \vdots \\ w_{m}X_{m} \end{array}\right].$$

**Substep 4**

Run the global test on the weighted gene expression matrix*wX* of the pathway and obtain *P*-value.

**Substep 5**

If the *P* value of the weighted gene expression matrix *wX*obtained in Substep 4 is smaller than the current min*P*, update the min*P* with this *P* value and update the optimal weight vector *optW* with the new *w* = [*w*_1_, *w*_2_,…, *w*_*m*_] constructed in Substep 2.

**End (for loop)**

Of course, the larger number of iteration increases the quality of the solution, but at the cost of higher computation time. We should also note that this weighting scheme assigns the weight to each gene across all samples as *absT* and *Qdiff *schemes do.

#### 2.3.4. *RWM* Based on Random Weight Matrices Generated by a Computational Approach

In contrast to the three schemes assigning the same weight across all samples for a gene, *RWM *(*random weight matrix*) scheme assigns different weights to all samples for a gene. Essentially, *RWM* scheme uses the same algorithm of *RWV* scheme except that it generates *n* × *m* random values instead of *m* random values, for the *n* samples in the pathway of *m* genes. The *n* × *m* random values in the predefined range are multiplied to the *n* × *m* gene expression data. Among all sets of random weights it applied, the scheme selects an optimal set of weights that minimizes the *P*-value in the global test, or the OOB error rate in the random forests for the pathway. The weighted gene expression matrix *wX* of a pathway is expressed in the following matrix:

(7) $$wX=\left[\begin{array}{ccccc} w_{1,1}X_{1,1} & w_{1,2}X_{1,2} & ... & & w_{1,m}X_{1,m}\\ w_{2,1}X_{2,1} & ... & ... & & w_{2,m}X_{2,m}\\ ... & ... & ... & & ...\\ w_{n-1,1}X_{n-1,1} & ... & ... & & w_{n-1,m}X_{n-1,m}\\ w_{n,1}X_{n,1} & w_{n,2}X_{n,2} & ... & & w_{n,m}X_{n,m} \end{array}\right].$$

Obviously, *RWM* scheme can find a better solution in minimizing the *P*-value or the OOB error than *RWV* scheme, but it is computationally more complex.

### 2.4. Datasets

**Real Datasets**

The first real dataset we used for our study is the well-known type II diabetes microarray gene expression dataset obtained from Mootha et al. [28], consisting of 278 pathways for 13,842 genes, sampled from 26 people with type II diabetes and 17 without. The pathways were obtained from KEGG pathway database (http://www.genome.jp/kegg/pathway.html), and the curate pathways were constructed from known biological experiments performed by Mootha et al. Another real dataset we used is the canine dataset obtained from Enerson et al. [29], consisting of 441 pathways for 6,592 genes, sampled from 12 dogs with lesion and 17 without. The canine dataset was generated from the investigative toxicology studies designed to identify the molecular pathogenesis of a drug-induced vascular injury in coronary arteries of dogs, which were treated with adenosine receptor agonist CI-947. The canine genes were mapped to human orthologs, and the human orthologs for dogs were generated by matching the genes sequence using *BLASTx* [13, 29]. Note that not all genes in a pathway have the same significant relevancies to the related disease. Some genes in a pathway could be related more significantly to the disease and some genes less or not at all. The pathway ID 36 in the type II pathway dataset, for instance, contains several genes such as CAP1, MAPP2K6, ARF6, and SGK, which are known to be related to the human insulin signaling, while containing other genes whose relevancies to the type II diabetes are not known yet [21].

**Simulated Datasets**

To study the weighting effect with more control, we created two simulated datasets using the simulator function available in the boost R package, which allows a simulated data to retain the same mean and the same correlation structure of the original pathway data [13, 36]. As the basis of our simulations, we selected two real pathways containing more than 20 genes and generating high *P* value in the global test or high OOB error rate in the random forests under uniform weight, to manifest the weighting effect more clearly. One pathway is “MAP00480_Glutathione_metabolism,” ID 164 from the type II diabetes dataset, containing 26 genes, ranked in the 277th with *P*-value 0.95 in the global test. Another pathway is “Eicosenoid Metabolism,” ID 441 from the canine dataset, containing 21 genes, ranked in the 421st with out-of-bag (OOB) error rate 0.48% in the random forests. For both cases, we used the multivariate normal distribution to create the simulated pathway data for sample size of 30, 50, and 100, with normal and disease group assigned with even number of samples.

## 3. Results and Discussion

We applied each proposed weighting scheme on each dataset in the global test and ranked the pathways in the increasing order of *P* values obtained from the global test. From the ordered list of pathways for each output set, we selected the top 20 pathways for our analysis. In the random forests case, we only applied *RWM* scheme, since the other three proposed schemes apply the same weight across all samples for genes, and that does not change the outcome of the out-of-bag error calculations for the genes by the random forests algorithm. For the random forest application results, we selected top 33 pathways instead of 20, in the increasing order of OOB error rates, to include the multiple pathways tied in some ranks within the 20th. Ranking pathways is important in the pathway analysis because it enables researchers to focus on a small number of pathways, which are estimated as statistically significant in terms of the relationship to the disease or phenotype of interest. In this paper, we focus on the top 20 or 33 selected pathways groups for each weighting scheme for the performance analysis of the proposed schemes and the comparison of them to the performance of uniform weight.

**Figure 1:**
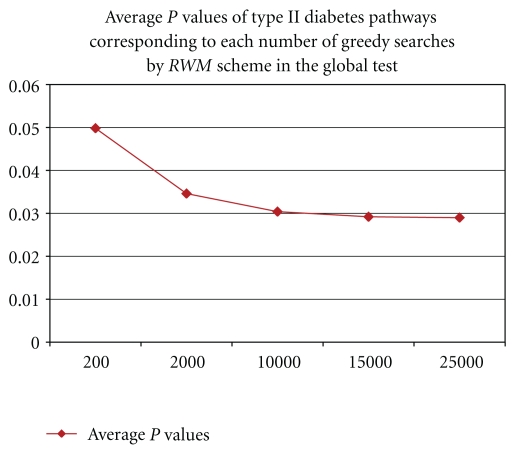
Average *P*-value of all Type II diabetes pathways versus number of iterations for *RWM* in the global test.

For the greed search for the optimal set of weights in the applications of *RWV *and *RWM* schemes, we used 25,000 iterations, since our experiments on the type II diabetes dataset in the global test showed no meaningful decrease in the *P* values, for the iterations of 20,000 or greater. The average *P*-values of the type II diabetes pathways corresponding to different number of iterations for running *RWM* scheme in the global test are displayed in Figure 1. 

To help readers refresh the memory of our four proposed weighting schemes before we discuss the application results of those in the following sections, we provide a brief summary of the four schemes in Table 1.

### 3.1. The Global Test Application Results

#### 3.1.1. Reduction of *P* Values

**Type II Diabetes Dataset**

**Figure 2:**
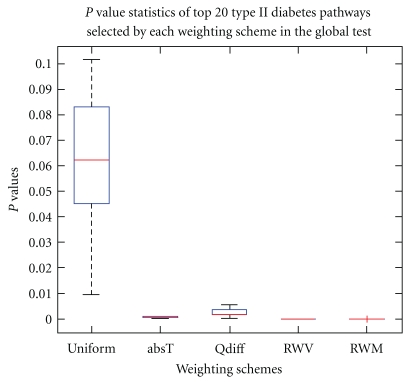
*P*-value distributions for the top 20 Type II diabetes pathways selected by each weighting scheme in the global test.

The pathway identification numbers (PID) of the type II diabetes pathways in all top 20 groups are displayed in Table 2. While the average *P*-value of the 20 pathways under uniform weight is 0.0612, it is much smaller under the proposed weighting schemes. In terms of the *P* value reduction, *RWM* performed best (with the average *P*-value of.0001), followed by *absT* (.0007), *Qdiff* (.0027), and *RWV* (.0044). The amounts of reduction are ranged from.0611 for *RWM* to.0568 for *RWV*. As another metric to examine the impact of our weighting schemes, we counted the total number of pathways having *P*-value less than 0.05. Among all 278 pathways in the dataset, *RWM* yields the largest number (=264) of pathways with *P*-values less than.05, followed by *absT* (with 142), *Qdiff *(with 74), *RWV *(with 66), and uniform weight (with 8). Those results reveal that our schemes effectively reduce the *P*-values of the pathways compared to the uniform weight. The statistics of the *P*-value distributions for all 20 pathways groups are shown in the box plots in Figure 2. In terms of the *P* values, *RWM *is the best followed by *absT*, and uniform weight is the worst. The dispersion of *P* values for uniform weight is the widest among all with largest number of outliers.

**Canine Dataset**

**Figure 3:**
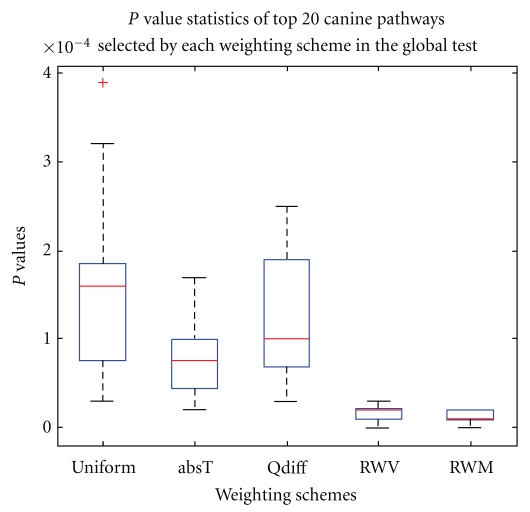
*P*-value distributions for the canine pathways selected by each weighting scheme in the global test.

According to the pathway analysis performed by Pang et al., the canine dataset has a relatively large number of differentially expressed genes [13]. We were interested in the performance of the proposed weighting schemes for such a dataset. The 20 pathways groups for all weighting schemes are displayed in Table 3. The average *P*-value of the 20 pathways for uniform weight is.00015, but it is also smaller for our weighting schemes. In terms of the *P* value reduction, the best performing scheme is *RWM* (with average *P*-value of.00001), followed by *absT* (.00002), *RWV* (.00002), and *Qdiff *(.00012). The reduction amounts are ranged from.00014 for *RWM* to 0.00003 for *Qdiff*. Compared to the type II diabetes pathways results, the reduction amount for the canine pathways are smaller. Such result is not unexpected, since the canine dataset is known to have more differentially expressed genes and may leave smaller room to improve. Among all 441 pathways in the dataset, *RWM* has the largest number (=431) of pathways having *P* value less than.05, followed by *absT* (with 405), *RWV* (with 388), uniform weight (with 204), and *Qdiff* (with 170). Our weighting schemes except *Qdiff* double the number of pathways with *P* values less than.05. It is rather interesting that *Qdiff* improves the *P* values of the 20 pathways over the uniform weight but decreases the number of total pathways with *P* values less than.05. The *P* value for all 20 pathways groups are shown in the box-plots in Figure 3. In terms of *P*-values, *RWM* and *RWV* are best followed by *absT*, and uniform weight and *Qdiff* are worst. The *P* values for *RWM* and *RWV* are similar, but *RWM *is better in terms of outliers.

**Simulated Datasets**

Upon our observation that *RWM* performs best in terms of *P*-value reduction, we applied *RWM* scheme on our simulated data to study the *P* value reduction in a more controlled environment. The *P* values of all simulated pathway data under uniform weigh and *RWM* scheme are given in Table 4. In the simulation case 1, the *P* values of the simulated pathways with 26 genes for 30, 50, and 100 samples were.2246,.2155, and.2573, respectively, under uniform weight (in Table 4(a)), but reduced to.0014,.0007, and.0002, respectively, under *RWM* scheme (in Table 4(b)). In the simulation case 2, the *P* values of the simulated pathways with 21 genes for 30 and 50 samples were.0289 and.0004 under uniform weight, but.0002 and.0001, respectively under *RWM* scheme. However, for the sample size 100 data, the *P*-value was zero under uniform weight, and no further improvement was by the *RWM*.

#### 3.1.2. Change of Ranks and New Significant Pathways

Our weighting schemes reduce *P* values of most pathways in each dataset and hence change the ranks of the pathways determined by the uniform weight. So, some pathways in low ranks under the uniform weight improve their rankings and may draw researchers' attention. We describe a few such cases in the following.

**Type II Diabetes Dataset**

We observed five pathways with pathway identification numbers (PIDs) of 13, 43, 51, 66, and 109 under *absT* scheme are originally ranked in the 107th or below under uniform weight. Interestingly, these pathways are reported to be associated with the type II diabetes in some ways in a couple of papers [37, 38]. The names, ranks, and *P* values of those pathways under uniform and *absT* scheme are given in Table 5. Such low ranking pathways might have been ignored by researchers under the uniform weight, while they would draw researchers' attention with our weighting schemes.

**Canine Dataset**

Six canine pathways with PIDs of 133, 154, 156, 320, 375, and 420 under *absT* scheme are originally ranked in the 258th or below under uniform weight. The associations of these new identified significant pathways to the cancer-related disease are also reported in several papers [39, 40, 40]. The names, ranks, and *P* values of those pathways under *absT* scheme are compared to those under uniform weight in Table 6. We observe similar impacts on pathway ranks induced by our other weighting schemes. They are not reported here to conserve space, but available in the first author's technical report.

#### 3.1.3. Overlapping Pathways

While those newly identified significant pathways under would draw researchers' fresh attentions, pathways identified as significant repeatedly under multiple weighting schemes may worth additional attention by researchers. We observed that several pathways hold high rankings across different weighting schemes, and their biological associations to the related diseases are discussed in numerous reports. We indicated those overlapping pathways appearing in three or more weighting schemes in bold faces in Tables 2 and 3. We discuss them in more detail for the two datasets in the following.

**Type II Diabetes Dataset**

Overlapping pathways among the top 20 groups include Alanine and aspartate metabolism (PID = 4), Glutamate metabolism (PID = 92), MAP00252 Alanine and aspartate metabolism (PID = 140), MAP00430 Taurine and hypotaurine metabolism (PID = 158), Oxidation Phosphorylation (PID = 228), and presented in bold faces in Table 2. Among them, oxidation phosphorylation (PID = 228) and glutamate metabolism (PID = 92) are well known type II diabetes pathways[28, 31].Alanine and aspartate metabolism (with PID = 4), glutamate metabolism (PID = 92), MAP00430_Taurine_and_hypotaurine_metabolism (PID = 158), MAP00252_Alanine_and_aspartate_ metabolism (PID = 140), and Alanine and aspartate metabolism (PID = 4) are also reported to be strongly associated with the type II diabetes in some ways by some researchers [31, 41, 42]. It is interesting to notice that pathways of PIDs 4 and 140 retain the high ranks (4th or above) across three different schemes.

**Canine Dataset**

Androgen and estrogen metabolism (PID = 17), tryptophan metabolism (PID = 39), multistep regulation of transcription by Pitx (PID = 117), RNA polymerase III transcription (PID = 151), mitochondrial carnitine palmitoyltransferase system (PID = 217), and Rho cell motility signaling pathway (PID = 391) are overlapping among different weighting schemes. Among them, tryptophan metabolism (PID = 39) and mitochondrial carnitine palmitoyltransferase system (PID = 217) hold the 8^th^ or higher ranks, and the biological significance of the two pathways to lesions or cancerous lesions are discussed by many researchers [32–34, 43–46, 46]. Biological associations of the other overlapping pathways to the related disease are also discussed in some reports [47, 48, 48].

#### 3.1.4. Prediction Performances

Prediction rates are another metric we used to measure the performances of our weighting schemes. Using LDA (linear discriminator analysis), SVML (support vector machine with a linear kernel), SVMP (support vector machine with a polynomial kernel), and KNN (k-nearest neighbors) classification methods, we measured the prediction performance of all genes in a pathway and take the average of it for all pathways in those 20 groups and cross validated those classification results using the LOOCV (leave-one-out cross validation) technique. The prediction performances of the pathways in all 20 groups are presented in Tables 7 and 8 for the two datasets.

As we can see in the Tables 7 and 8, however, the predicting power of those pathways selected under the proposed weighting scheme (except *RWM*) shows insignificant difference between those selected under uniform weight. This explains that those classifiers we used for the performance measurement are single gene based and do not consider gene's dependencies in the pathway. Since our weighting schemes, except *RWM*, apply the same weight across all groups of samples for each gene, the classifying power of the genes do not change. Hence, those classifiers cannot be used to evaluate the improvement of predicting power of the pathways selected using our weighting schemes. Note that unlike other schemes, *RWM* applies different weights to all samples for a gene, and thus the classifiers measure the weighting effect on the samples for each gene but not on the genes in the pathways. We discuss the improvement of predicting power only for the 20 pathways selected under *RWM* scheme. 

Table 6 shows the improvement of the predicting power of the genes in the 20 type II diabetes pathways selected under *RWM* scheme. The prediction rate 0.5 measured by LDA for 20 pathways for uniform weight was increased to 0.81 for *RWM*, which is 24% improvement. The prediction improvement made by *RMW* scheme was 18% when measured by SVML, 23% by SVMK, and 21% by KNN. As for the canine dataset results, the improvements were 2%, 0%, −1%, and 3% as measured by LDA, SVML, SVMP, and KNN, respectively, as shown in Table VIII. The small improvement for the canine pathways compared to that for the type II pathways may share the same reason with the small reductions of the *P*-values: the canine dataset to have relatively more differentially expressed genes, and thus may leave smaller room to improve.

### 3.2. Random Forests Results

The proposed* absT* and *Qdiff* weighting schemes are designed to incorporate into the covariance structure of the random effect R when the test statistic *Q* is calculated in the global test for a group of genes. Hence, the application of such schemes in the random forests method is not appropriate, and indeed the poor experimental results confirmed it. *RWV* application in the random forests is not appropriate either, since it assigns the same weight across all samples for a gene like *absT* and *Qdiff* schemes. Thus, we only discuss the application results of *RWM* scheme in the random forests method case, and compare them with those of uniform weight. We also compare those to the *RWM* application results in the global test method.

#### 3.2.1. Reduction of out-of-Bag (OOB) Error Rate

The out-of-bag (OOB) error rate is the percentage of time that the random forests classification or regression is incorrect for the OOB data. To obtain an unbiased estimate of the classification or regression error in the random forests, OOB data run down the tree, and the overall error rate is computed when a specified number of trees are added to the forest. We used 50,000 trees to estimate the classification error, the same number used in the similar experiments performed by Pang et al. for their pathway analysis using the random forests method [13, 25–27].

**Type II Diabetes Dataset**

Table 9 displays the PIDs and the OOB error rates of top 33 type II diabetes pathways in the random forests under uniform weight and *RWM* scheme. While the average OOB error rate under uniform weight is 35%, it is only 18% under *RWM* scheme. The OOB error rate is reduced into almost a half by the application of *RWM* scheme in the random forests.

**Canine Dataset**

The average error rate 8% under uniform weight is reduced to 6% under *RWM* scheme, which is only a half of the reduction made for the type II diabetes data under *RFM*.  Table 10 shows the PIDs and the OOB error rates of the 33 canine pathways under uniform weight and *RWM* scheme. Again, a larger number of differentially expressed genes in the canine dataset may leave only a small room for weighting to improve the application result.

**Simulation Datasets**

In the simulation case 1, the error rates of the simulated pathways with 26 genes for 30, 50, and 100 samples are 0.27, 0.48, and 0.30, respectively, under uniform weight and reduced significantly to 0.13, 0.36, and 0.22, respectively, under *RWM* scheme. In the simulation case 2, the error rates of the simulated pathways with 21 genes for 30 and 50 samples are 0.50 and 0.30, respectively, under uniform weight and reduced to 0.30 and 0.20 under *RWM,* respectively. For the sample size of 100, the error rates were same 0.24 for both uniform and *RWM* schemes. The OOB error rates of simulated pathways under uniform and *RWM* scheme are given in Table 11. The substantial reduction of the error rates under *RWM* scheme over uniform weight in the two simulation cases supports our hypothesis that applying different weights to genes in the pathway analysis may enhance the quality of the analysis.

#### 3.2.2. Change of Ranks and New Significant Pathways

Fourteen type II diabetes and five canine pathways out of each 33 group selected under *RWM* scheme are originally ranked in the 100th or below under uniform weight. We list each five most significantly changed type II diabetes and canine pathways in Tables 12 and 13, respectively, to compare their original ranks under uniform weight to the new ranks under *RWM* scheme

#### 3.2.3. Overlapping Pathways

Three pathways with PIDs of 1, 4, and 140 from the type II diabetes dataset overlap between uniform weight and *RWM* scheme. Nine canine pathways with PIDs of 17, 39, 117, 151, 274, 354, 368, 378, and 395 for the canine dataset overlap. Further, several pathways overlap between the global test and the random forests application results both under *RWM* scheme. Four type II diabetes pathways with PIDs of 144, 176, 197, and 245 and five canine pathways with PIDs of 17, 39, 40, 117, and 274 are such pathways. Interestingly, the four canine pathways under *RWM* scheme overlapping between the global test and the random forests also overlap between uniform weight and *RWM* scheme in the random forests. We believe that such pathways overlapping across different weighting schemes applied in the same pathway analysis method, and across different pathway analysis methods for the same weighting scheme, may have even stronger relevancies to the related phenotypes.

#### 3.2.4. Prediction Performances

The prediction rates of each 33 pathways group for each real dataset are given in Table 14. According to the four classifiers we used to measure the prediction rates of the selected pathways, *RWM* scheme improved the prediction rate of the type II pathways from 52% to 63% (LDA), 48% to 64% (SVML), 49% to 54% (SVMP), and 52% to 59% (KNN). But for the canine pathways, it worsened the prediction rates. Presumably, weights applied to the genes of good predicting power in the significant canine pathways may add noises to the expression data of those genes and degrade the predicting power.

### 3.3. Biological Support

To investigate further the significance of the proposed weighting scheme in terms of biological meaning, we searched the functional annotations of the genes in the selected pathways using weights. We particularly sought the biological support for *absT* scheme, since our overall performance analysis on our four proposed schemes finds the *absT* is the most useful and efficient requiring no complex computation like *RWM* scheme. Using DAVID functional annotation tool [49], we extracted 952 Homo Sapiens genes from 2,150 genes contained the 20 pathways selected under *absT* scheme. DAVID tool identified eleven enriched genes associated with type II diabetes with *P*-value.01 (by the gene-disease association search with GENTIC_ASSOCIATION_DB_DISEASE option). We list those eleven genes in Table 15. 

Interestingly enough, the DAVID tools failed to identify any enriched genes for the type II diabetes in the top 20 pathways selected under uniform weight.

## 4. Conclusions

In this paper, we proposed to apply different weighting schemes in pathway-based analysis, based on our intuitive thought that genes more differentially expressed between two different groups of samples (normal versus tumor samples) will contribute more significantly to the related biological function or disease. We devised four weighting schemes *absT*, *Qdiff*, *RWV*, and *RWM *that assign different weights to genes in the pathways. The former two schemes assign weights to genes based on their relevancy to the related disease, and the latter two schemes select the weights minimizing *P*-values or error rates among all sets of weights randomly assigned. We investigated the weighting impact in the pathway-based analysis using two real and two simulated pathway datasets. To our best knowledge, we are the first team to apply weights to genes in the pathway-based analysis in open literature.

We made a few interesting observations through our investigations. First, our weighting schemes effectively reduce *P*-values of the pathways in the global test and OOB error rates in the random forests for all datasets used in our experiments. Second, our schemes increase the number of pathways with *P*-values less than 0.05. *RWM* performs best among all proposed schemes in terms of *P*-value and OOB error rate reduction, but the scheme is computationally expensive. Third, *RWM* improves prediction rates of high ranking pathways. Fourth, all the improvements discussed above are more significant for the type II diabetes dataset than the canine dataset. It may be due to the fact that genes with better predicting power or more differentially expressed leave less room for further improvement. In addition to the above improvements, our schemes could find potentially significant pathways which were missed by uniform weight. As described in Section 3, pathways whose ranks improved by weighting are associated to the related diseases according to the reports presented in numerous literatures. Finally, it is worth noting that *absT* and *Qdiff* schemes are, in theory, inferior to *RWM* scheme, but are computationally far less complex than *RWM*. So, it may be a good idea to apply them in case one as they cannot afford large computing power or long computing time. 

We have unresolved issues for evaluating the weighting effect in the prediction performance of our proposed schemes. The four prediction methods (LDA, SVML, SVMP, and KNN) are single gene based and cannot be used to evaluate our schemes *absT*, *Qdiff,* and *RWV*. In the perspective of those methods, the same weight assigned across all samples of a single gene does not make any change in terms of classifying two different groups of samples for that single gene. Even for *RWM* scheme, they only can evaluate the weighting effect on samples but not on genes, since they cannot consider the interactive relationship or dependencies among genes in a group. It is necessary to develop a new prediction method that considers the dependencies among genes for more accurate assessment of weighting effects in the pathway-based analysis. Developing such prediction method is left for future research.

## Figures and Tables

**Table 1:** A brief summary of the four proposed weighting schemes. The algorithms are expressed for the pathway of *m* genes and *n* samples. *absT* and *Qdiff* algorithms calculate the weight for the *j*_th_ gene in a pathway, and *RWV* selects an optimal random weight vector *w* minimizing *P*-value or OOB error rate, and *RWM* selects an optimal weight matrix *w* minimizing *P*-value or OOB error rate.

Name	Algorithm	Notes
*absT*	*W* ^|*T*|^_(*j*)_ = |*T*_(*j*)_|/∑_*j*=1_^*m*^|*T*_(*j*)_|	Based on two-sample *t* statistics of a gene, apply the same weight across all samples of a gene
*Qdiff*	*W* ^|*Q*_*diff*_|^_(*j*)_ = |*Q* − *Q*_(−*j*)_|/∑_*j*=1_^*m*^|*Q* − *Q*_(−*j*)_|	Based on the global test statistic *Q* for a pathway, apply the same weight across all samples for a gene
*RWV* (*Random Weight Vector*)	$wX=\left[\begin{array}{c} w_{1}X_{1}\\ w_{2}X_{2}\\ \vdots \\ w_{m}X_{m} \end{array}\right]$	*m* number of random weights in predefined range that minimizes *P*-value in the global test, or OOB error rates in the random forests for a pathway
*RWM* (*Random Weight Matrix*)	$wX=\left[\begin{array}{cccc} w_{1,1}X_{1,1} & w_{1,2}X_{1,2} & ... & w_{1,m}X_{1,m}\\ w_{2,1}X_{2,1} & ... & ... & w_{2,m}X_{2,m}\\ ... & ... & ... & ...\\ w_{n-1,1}X_{n-1,1} & ... & ... & w_{n-1,m}X_{n-1,m}\\ w_{n,1}X_{n,1} & w_{n,2}X_{n,2} & ... & w_{n,m}X_{n,m} \end{array}\right]$	*m* × *n* number of random weights in predefined range that minimizes *P*-value in the global test or error rates in the random forests for a pathway

**Table 2:** Top 20 type II diabetes pathways selected in the global test under each weighting scheme (PID stands for pathway identification number).

	Weighting schemes									
	*Uniform*		*absT*		*Qdiff*		*RWV*		*RWM*	
Ranks	PID	*P*-value	PID	*P*-value	PID	*P*-value	PID	*P*-value	PID	*P*-value
1	**158**	.0098	40	.0002	**140**	.0004	**140**	.0003	195	.0000
2	264	.0114	**140**	.0003	57	.0008	**92**	.0006	172	.0000
3	**140**	.0218	13	.0003	41	.0015	139	.0009	127	.0001
4	**4**	.0331	37	.0004	**4**	.0017	**4**	.0011	2	.0001
5	**228**	.0431	57	.0005	264	.0018	264	.0015	76	.0001
6	168	.0474	44	.0006	**158**	.0018	**158**	.0015	235	.0001
7	73	.0503	**92**	.0006	20	.0018	157	.0022	199	.0001
8	139	.0509	278	.0006	157	.0018	20	.0024	198	.0001
9	204	.0555	66	.0007	193	.0018	**228**	.0028	261	.0001
10	**92**	.0618	56	.0007	26	.0019	203	.0035	277	.0001
11	162	.0625	51	.0007	**92**	.0027	26	.0048	**158**	.0001
12	203	.0746	109	.0007	232	.0035	17	.0049	80	.0001
13	229	.0784	139	.0008	37	.0036	193	.0064	263	.0001
14	201	.0817	104	.0009	58	.0040	73	.0066	19	.0001
15	120	.0823	**4**	.0010	59	.0040	208	.0069	165	.0001
16	76	.0831	217	.0010	60	.0040	8	.0073	42	.0001
17	128	.0847	110	.0010	61	.0040	79	.0077	144	.0001
18	274	.0937	43	.0010	62	.0040	16	.0078	162	.0001
19	247	.0968	36	.0011	63	.0040	252	.0090	258	.0001
20	22	.1015	**228**	.0011	139	.0049	173	.0093	1	.0001

Total *P*-values		1.2244		.0142		.0540		.0875		.0018
Average *P*-value		.0612		.0007		.0027		.0044		.0001

**Table 3:** Top 20 canine pathways selected in the global test under each weighting scheme (PID stands for pathway identification number).

	Weighting schemes									
	*Uniform *		*absT *		*Qdiff*		*RWV *		*RWM*	
Ranks	PID	*P*-value	PID	*P*-value	PID	*P*-value	PID	*P*-value	PID	*P*-value
1	**117**	.00003	**151**	.00000	368	.00003	**17**	.00000	**39**	.00000
2	**39**	.00004	**391**	.00000	**39**	.00004	**391**	.00000	**40**	.00000
3	394	.00004	326	.00001	**217**	.00006	368	.00001	223	.00001
4	**217**	.00007	360	.00001	394	.00007	**117**	.00001	160	.00001
5	304	.00007	73	.00001	73	.00007	**151**	.00001	295	.00001
6	183	.00008	**117**	.00002	304	.00007	202	.00001	304	.00001
7	**40**	.00009	**39**	.00002	247	.00007	**39**	.00001	283	.00001
8	440	.00009	156	.00002	**40**	.00008	175	.00001	**217**	.00001
9	159	.00011	133	.00002	440	.00008	210	.00002	387	.00001
10	**17**	.00015	**217**	.00002	**117**	.00010	247	.00002	64	.00001
11	**151**	.00017	94	.00002	183	.00010	394	.00002	421	.00001
12	45	.00017	375	.00002	157	.00010	239	.00002	135	.00002
13	**391**	.00017	**40**	.00002	64	.00011	45	.00002	129	.00002
14	368	.00018	261	.00002	159	.00015	100	.00002	374	.00002
15	192	.00018	192	.00002	45	.00018	310	.00002	165	.00002
16	261	.00019	154	.00002	261	.00020	326	.00002	183	.00002
17	87	.00025	157	.00003	**151**	.00020	304	.00003	265	.00002
18	422	.00028	420	.00003	**391**	.00020	281	.00003	20	.00002
19	223	.00032	320	.00003	**17**	.00020	360	.00003	397	.00002
20	354	.00039	422	.00003	192	.00025	336	.00003	**17**	.00002

Total *P*-values		.00307		.00037		.00236		.00034		.00027
Average *P*-value		.00015		.00002		.00012		.00002		.00001

**Table:** (a) Simulation Case 1.

No. of samples	No. of genes	No. of tested	Statistic *Q *		Expected *Q *	sd-of-*Q *		*P* values	
			*Unif*	*RWM*		*Unif*	*RWM*	*Unif*	*RWM*
30	26	26	13.51	27.68	10	7.64	3.96	0.2246	0.0014
50	26	26	13.42	27.49	10	6.59	3.61	0.2155	0.0007
100	26	26	12.33	27.44	10	6.60	3.25	0.2573	0.0002

**Table:** (b) Simulation Case 2.

No. of samples	No. of genes	No. of tested	Statistic *Q *		Expected *Q *	sd-of-*Q *		*P* values	
			*Unif*	*RWM*		*Unif*	*RWM*	*Unif*	*RWM*

30	21	21	34.60	46.51	10	9.46	5.57	0.0289	0.0002
50	21	21	66.41	56.91	10	7.94	5.87	0.0004	0.0001
100	21	21	97.11	70.69	10	8.03	5.42	0.0000	0.0000

**Table 5:** Five new significant type II diabetes pathways selected in the global test under *absT *scheme: *P* values and ranks of them are compared to those under uniform weight.

Pathway name	*P*-values		Ranks	
	*Uniform*	*absT*	*Uniform*	*absT*
(PID = 13) Apoptosis	.3658	.0003	111	2

(PID = 66) Cell cycle	.4871	.0007	155	9

(PID = 51) c3_U133_probes	.3822	.0007	116	9

(PID = 109) Integrin-mediated cell adhesion	.3876	.0007	119	9

(PID = 43) c22_U133_probes	.3599	.0010	107	15

**Table 6:** Seven new significant canine pathways selected in the global test under* absT *scheme: *P* values and ranks of them are compared to those under uniform weight.

Pathway name	*P*-values		Ranks	
	*Uniform*	*absT*	*Uniform*	*absT*
(PID = 133) Activation of Csk by cAMP-dependent protein kinase inhibits signaling through the T cell receptor	.1006	.00002	258	6

(PID = 156) Steps in the glycosylation of mammalian N-linked oligosaccarides	.1308	.00002	278	6

(PID = 375) PTEN-dependent cell cycle arrest and apoptosis	.4451	.00002	371	6

(PID = 154) TPO signaling pathway	.5504	.00002	389	6

(PID = 420) Trefoil factors initiate mucosal healing	.2226	.00003	330	17

(PID = 320) CDK regulation of DNA replication	.5563	.00003	390	17

**Table 7:** Prediction rates of top 20 type II diabetes pathways selected in the global test under each weighting scheme.

Prediction methods	*Uniform*	*absT*	*Qdiff*	*RWV*	*RWM*
LDA	0.57	0.58	0.53	0.55	0.81
SVML	0.61	0.59	0.58	0.61	0.79
SVMP	0.51	0.53	0.51	0.53	0.74
KNN	0.55	0.68	0.53	0.59	0.76

**Table 8:** Prediction rates of top 20 canine pathways selected in the global test under each weighting scheme.

Prediction methods	*Uniform*	*absT*	*Qdiff*	*RWV*	*RWM*
LDA	0.84	0.82	0.86	0.86	0.86
SVML	0.86	0.86	0.86	0.86	0.86
SVMP	0.71	0.70	0.72	0.72	0.70
KNN	0.84	0.83	0.83	0.84	0.87

**Table 9:** Top 33 type II diabetes pathways selected in the random forests under uniform weight and *RWM* scheme.

Index	*Uniform* weight				*RWM *scheme			
	Rank	*PID*	No. of genes	OOB (%)	Rank	PID	No. of genes	OOB (%)
1	1	79	33	0.26	1	**140**	22	0.11
2	2	**4**	18	0.29	2	113	26	0.14
3	2	**140**	22	0.29	2	192	1	0.14
4	4	36	116	0.31	4	106	5	0.17
5	4	124	4	0.31	5	117	26	0.17
6	4	230	121	0.31	4	**144**	7	0.17
7	7	**5**	6	0.34	4	163	8	0.17
8	7	16	49	0.34	4	164	26	0.17
9	7	32	157	0.34	4	176	3	0.17
10	7	46	36	0.34	4	197	11	0.17
11	7	51	185	0.34	4	235	3	0.17
12	7	109	91	0.34	4	244	46	0.17
13	7	141	4	0.34	4	245	11	0.17
14	7	229	133	0.34	4	250	12	0.17
15	7	267	4	0.34	4	251	1	0.17
16	16	**1**	2	0.37	4	254	25	0.17
17	16	6	3	0.37	4	274	16	0.17
18	16	**11**	15	0.37	4	275	9	0.17
19	16	13	92	0.37	19	**1**	2	0.20
20	16	37	235	0.37	19	**4**	18	0.20
21	16	40	240	0.37	19	**5**	6	0.20
22	16	49	188	0.37	19	**11**	15	0.20
23	16	59	194	0.37	19	24	14	0.20
24	16	76	3	0.37	19	42	2	0.20
25	16	**144**	7	0.37	19	52	122	0.20
26	16	162	2	0.37	19	69	20	0.20
27	16	173	11	0.37	19	74	1	0.20
28	16	194	13	0.37	19	78	6	0.20
29	16	201	19	0.37	19	80	5	0.20
30	16	207	3	0.37	19	85	8	0.20
31	16	209	21	0.37	19	86	39	0.20
32	16	227	13	0.37	19	98	71	0.20
33	16	228	43	0.37	19	99	13	0.20

Total			2050	11.29			578	5.86
Average			64	0.35			18	0.18

**Table 10:** Top 33 canine pathways selected in the random forests under uniform weight and *RWM* scheme.

Index	*Uniform* weight				*RWM* scheme			
	Rank	PID	No. of genes	OOB (%)	Rank	PID	No. of genes	OOB (%)
1	1	**274**	4	0.03	1	**39**	9	0.00
2	1	**354**	6	0.03	2	**17**	8	0.03
3	1	**378**	15	0.03	2	45	40	0.03
4	1	**395**	14	0.03	2	182	5	0.03
5	5	**17**	8	0.07	2	220	7	0.03
6	5	**73**	5	0.07	2	**274**	4	0.03
7	5	**100**	18	0.07	2	**289**	13	0.03
8	5	**151**	8	0.07	2	**354**	6	0.03
9	5	**239**	9	0.07	2	**368**	19	0.03
10	5	287	15	0.07	2	**378**	15	0.03
11	5	**330**	14	0.07	2	**395**	14	0.03
12	5	339	19	0.07	2	440	59	0.03
13	5	349	68	0.07	13	24	7	0.07
14	5	**368**	19	0.07	13	40	4	0.07
15	15	**39**	9	0.10	13	59	14	0.07
16	15	89	22	0.10	13	**73**	5	0.07
17	15	**117**	10	0.10	13	**100**	18	0.07
18	15	129	7	0.10	13	**117**	10	0.07
19	15	147	11	0.10	13	**151**	8	0.07
20	15	**156**	4	0.10	13	154	16	0.07
21	15	171	12	0.10	13	**156**	4	0.07
22	15	173	46	0.10	13	162	18	0.07
23	15	175	34	0.10	13	**202**	27	0.07
24	15	**202**	27	0.10	13	204	10	0.07
25	15	**223**	4	0.10	13	**223**	4	0.07
26	15	230	3	0.10	13	229	6	0.07
27	15	**280**	17	0.10	13	234	8	0.07
28	15	281	32	0.10	13	**239**	9	0.07
29	15	**289**	13	0.10	13	264	15	0.07
30	15	**326**	11	0.10	13	269	7	0.07
31	15	380	25	0.10	13	**280**	17	0.07
32	15	391	7	0.10	13	**326**	11	0.07
33	15	436	6	0.10	13	**330**	14	0.07

Total			522	2.79			431	1.83
Average			16	0.08			13	0.06

**Table 11:** Random forests results for simulated datasets under uniform weight and *RWM* scheme: (a) simulation case 1 uses covariance structure and mean of Pathway ID 164 from type II diabetes dataset, (b) simulation case 2 uses covariance structure and mean of Pathway ID 441 from canine dataset.

No. of samples		(a) Simulation Case 1			(b) Simulation Case 2	
	No. of *genes *	OOB (%)		No. of genes	OOB (%)	
		*Uniform*	*RWM*		*Uniform*	*RWM*
30	26	0.27	0.13	21	0.50	0.33
50	26	0.48	0.36	21	0.30	0.20
100	26	0.30	0.22	21	0.24	0.24

**Table 12:** Type II diabetes pathways whose ranks are significantly changed under *RWM *in the random forests.

Pathway ID and name	Ranks		OOB (%)	
	*Uniform*	*RWM*	*Uniform*	*RWM*
PID 113 Limonene and pinene degradation	184	2	0.54	0.14
PID 106 Inositol metabolism	259	4	0.69	0.17
PID 164 MAP00480_Glutathione_metabolism(user defined)	242	4	0.63	0.17
PID 176 MAP00550_Peptidoglycan_biosynthesis(user defined)	259	4	0.66	0.17
PID 235 Peptidoglycan biosynthesis	259	4	0.66	0.17

**Table 13:** Canine pathways whose ranks are significantly changed under *RWM *in the random forests.

Pathway ID and name	Ranks		OOB (%)	
	*Uniform*	*RWM*	*Uniform*	*RWM*
PID 24 Alanine and aspartate metabolism	319	13	0.34	0.07

PID 59 Glycerolipid metabolism	242	13	0.28	0.07

PID 204 Role of PI3K subunit p85 in regulation of actin organization and cell migration	281	13	0.31	0.07

PID 229 Induction of apoptosis through DR3 and DR4/5 death receptors	157	4	0.21	0.07

PID 269 Ghrelin: regulation of food intake and energy homeostasis	188	4	0.28	0.07

**Table 14:** Prediction rates of top 33 canine pathways selected in the random forest under uniform weight and *RWM* scheme.

Prediction methods	Type II diabetes dataset		Canine dataset	
	*Uniform *	*RWM*	*Uniform*	*RWM*
LDA	0.54	0.63	0.87	0.78
SVML	0.53	0.64	0.88	0.84
SVMP	0.46	0.54	0.76	0.72
KNN	0.54	0.59	0.80	0.73

**Table 15:** Eleven genes associated with type II diabetes in the top 20 pathways selected under *absT* scheme.

Gene symbols	Gene names
CD36	cd36 antigen (collagen type i receptor, thrombospondin receptor)
CAS	Caspase 9, apoptosis-related cysteine peptidase
GPX3	Glutathione peroxidase 3 (plasma)
GSTT1	Glutathione s-transferase theta 1
SOD1	Superoxide dismutase 1, soluble (amyotrophic lateral sclerosis 1 (adult))
TPMT	Thiopurine s-methyltransferase
GSTM1	Glutathione s-transferase m1
CYP2E1	Cytochrome p450, family 2, subfamily e, polypeptide 1
LPL	Lipoprotein lipase
TNF	Lipoprotein lipase
GYS	Tumor necrosis factor (tnf superfamily, member 2)
